# Epidemiology, molecular characterisation and antimicrobial susceptibility of *Neisseria gonorrhoeae* isolates in Madrid, Spain, in 2016

**DOI:** 10.1017/S095026881900150X

**Published:** 2019-09-24

**Authors:** M. D. Guerrero-Torres, M. B. Menéndez, C. S. Guerras, E. Tello, J. Ballesteros, P. Clavo, T. Puerta, M. Vera, O. Ayerdi, J. C. Carrio, I. Mozo, J. Del Romero, J. A. Vázquez, R. Abad

**Affiliations:** 1 Neisseria Reference Laboratory, National Centre for Microbiology, Instituto de Salud Carlos III, Majadahonda, Madrid, Spain; 2 Centro Sanitario Sandoval, Instituto de Investigación Sanitaria San Carlos (IdISSC), Madrid, Spain

**Keywords:** Antimicrobial resistance, epidemiology, *Neisseria gonorrhoeae*, NG-MAST, risk factors

## Abstract

With the aim to elucidate gonococcal antimicrobial resistance (AMR)–risk factors, we undertook a retrospective analysis of the molecular epidemiology and AMR of 104 *Neisseria gonorrhoeae* isolates from clinical samples (urethra, rectum, pharynx and cervix) of 94 individuals attending a sexually transmitted infection clinic in Madrid (Spain) from July to October 2016, and explored potential links with socio-demographic, behavioural and clinical factors of patients. Antimicrobial susceptibility was determined by *E*-tests, and isolates were characterised by *N. gonorrhoeae* multi-antigen sequence typing. Penicillin resistance was recorded for 15.4% of isolates, and most were susceptible to tetracycline, cefixime and azithromycin; a high incidence of ciprofloxacin resistance (~40%) was found. Isolates were grouped into 51 different sequence types (STs) and 10 genogroups (G), with G2400, ST5441, ST2318, ST12547 and G2992 being the most prevalent. A significant association (*P* = 0.015) was evident between HIV-positive MSM individuals and having a ciprofloxacin-resistant strain. Likewise, a strong association (*P* = 0.047) was found between patient age of MSM and carriage of isolates expressing decreased susceptibility to azithromycin. A decrease in the incidence of AMR gonococcal strains and a change in the strain populations previously reported from other parts of Spain were observed. Of note, the prevalent multi-drug resistant genogroup G1407 was represented by only three strains in our study, while the pan-susceptible clones such as ST5441, and ST2318, associated with extragenital body sites were the most prevalent.

## Introduction

Sexually transmitted infection (STI) caused by *Neisseria gonorrhoeae* is a major public health problem with a high global incidence. In 2012, the World Health Organization (WHO) estimated 78 million new cases of gonorrhoea infections among adults worldwide [1].

Between 2009 and 2011, the first strains with high-level resistance to all extended-spectrum cephalosporins appeared in Japan, France and Spain [2–4]. To mitigate the spread of resistant strains, several clinical guidelines were published in 2012, 2015 and 2016 which recommended dual therapy with ceftriaxone 250–500 mg intramuscularly as a single dose plus azithromycin 1–2 g as single oral dose [5–7]. However, high rates of azithromycin resistance were reported [8] and the failure of dual therapy was observed in a number of studies [9–12].

In response to this emergent public concern, action plans to detect and prevent the emergence and spread of antimicrobial resistance (AMR) in *N. gonorrhoeae* have been recommended by both the WHO [13,14] and the European Centre for Disease Prevention and Control (ECDC) [15]. In this way, monitoring of strain populations by molecular techniques and determination of antimicrobial susceptibility of *N. gonorrhoeae* isolates became imperative, as well as gathering of data on clinical and social risk factors associated with AMR in order to identify at-risk patient groups and provide evidence-based data for the implementation of public health control programmes [16].

With the aim of further elucidating gonococcal AMR–risk factors, we carried out a retrospective analysis of the molecular epidemiology and antimicrobial susceptibility of *N. gonorrhoeae* isolates obtained from patients attending an STI reference clinic in Madrid (Spain) over a 4-month period in 2016, and potential links with socio-demographic, behavioural and clinical factors of the patients.

## Material and methods

### Patients and sample collection

This was a retrospective study among patients attending the STI outpatient clinic Centro Sanitario Sandoval, in Madrid. This centre works as a walk-in clinic and offers free and anonymous services to more than 30 000 patients/year. A total of 360 patient samples, positive for *N. gonorrhoeae* by PCR, between July and October 2016 were collected; 211 of these were also culture positive. A randomly selected panel of 104 *N. gonorrhoeae* isolates (cervix (*n* = 8), pharynx (*n* = 19), rectum (*n* = 35) and urethra (*n* = 42)) from 94 patients were included in the study.

Data on patient socio-demographic, behavioural and clinical factors were collected from standardised questionnaires and clinical histories.

The study was approved by the Ethics Committee of University Hospital La Princesa, in Madrid, Spain (reference number 2923*).

### Bacterial strains

All samples were cultured on Thayer-Martin medium and incubated for 48 h at 35–37 °C in a 5% CO_2_ atmosphere. *N. gonorrhoeae* isolates were identified by colony morphology, Gram staining, the oxidase test, biochemical tests (API NH; bioMérieux, Marcy-l'Etoile, France) and PCR (RealTime CT/NG; Abbot, Des Plaines, IL, USA). Isolates were stored at −80 °C in trypticase soy broth with 10% glycerol and transported to the reference laboratory at the National Centre for Microbiology (Institute of Health Carlos III, Spain) for antimicrobial susceptibility testing, serotyping and molecular characterisation.

### Antimicrobial susceptibility testing

The minimum inhibitory concentrations (MICs) of penicillin, ceftriaxone, cefixime, azithromycin, ciprofloxacin, tetracycline, gentamicin and spectinomycin were determined by *E*-test (bioMérieux) on GC agar base supplemented with 1% IsoVitalex, according to the manufacturer's instructions. MIC (mg/l) values were interpreted according to the European Committee on Antimicrobial Susceptibility Testing (EUCAST) and the Clinical and Laboratory Standards Institute (CLSI) 2016 breakpoints [17, 18]. All isolates were tested for penicillinase production using nitrocefin solution (Oxoid, Hampshire, UK). The ATCC *N. gonorrhoeae* strain 49 226 was used as an internal control.

### Serotyping and molecular characterisation

All isolates were serotyped with Phadebact^®^ Monoclonal GC Test (MKL Diagnostics, Sollentuna, Sweden) and characterised by the *N. gonorrhoeae* multi-antigen sequence typing (NG-MAST) system as previously described [19].

NG-MAST genogroups were defined by clusters of tightly associated sequence types (STs) that shared one of the two alleles with the other showing ⩾99% similarity (⩽5 bp difference for *por*B and ⩽4 bp for *tbp*B). Genogroups were designated G followed by the predominant ST within each group [20] or by using the lowest frequent ST when the genogroup had the same proportion of STs. For analysis of genogroups, the *por*B and *tbp*B sequences were concatamerised and their similarities determined using the maximum likelihood test. The tree was inferred using the Neighbor-Joining method by the Molecular Evolutionary Genetics Analysis version 7 (MEGA7) software [21].

### Statistical analysis

Categorical data were compared using *χ*^2^, Fisher exact or McNemar test, and continuous variables with Mann–Whitney *U* test. Determinants associated with AMR and/or decreased susceptibility (DS) were assessed by logistic regression analyses. Multivariable analysis was performed including all determinants associated in the univariable analysis (*P* < 0.1) and using backward selection. Statistical analysis was performed with SPSS statistics v23.0 and significance was set at *P* < 0.05.

## Results

A total of 211 *N. gonorrhoeae* isolates were collected in the study period; from these, a randomly selected panel of 104 isolates from 94 patients was studied further. Nine patients had *N. gonorrhoeae*-positive cultures in two anatomical sites (four pharynx and rectum, three pharynx and cervix, two pharynx and urethra), and one patient had two different episodes of gonorrhoea.

### Clinical and epidemiological data

The demographic and behavioural characteristics of the 94 study patients are summarised in Table 1. The median age was 30.5 years, 84 were male, 71 of which had sex with men (MSM) and 56 were of Spanish origin. The majority (61) reported being recreational drug users. The most commonly used drugs were: alcohol (excessive consumption 54.3%), poppers (25.5%), cannabis (18.1%), cocaine (16.0%) and *γ*-hydroxybutyric acid (14.9%); other narcotics accounted for <10% of users.

**Table 1.** Demographic and behavioural characteristics of the study population

Epidemiological characteristics (*n* = 94)	
Gender, *n* (%)	
Male	84 (89.4)
Female	10 (10.6)
Median age, years (range)	30.5 (18–51)
Age group, *n* (%)	
18–28	39 (41.5)
29–39	31 (33.0)
40–51	24 (25.5)
Origin, *n* (%)	
European	64 (68.1)
Latin American	23 (24.5)
American	2 (2.1)
Asian	5 (5.3)
Education Level, *n* (%)	
Elementary	3 (3.2)
Secondary	14 (14.9)
Higher	35 (37.2)
Unknown	42 (44.7)
Sexual behaviour, *n* (%)	
MSM	71 (75.5)
Heterosexual	20 (21.3)
Bisexual	3 (3.2)
Sex workers, *n* (%)	
Yes	9 (9.6)
No	85 (90.4)
*Toxic habits*	
IDU, *n* (%)	
Yes	2 (2.1)
No	92 (97.9)
Recreational drug users, *n* (%)	
Yes	61 (64.9)
No	20 (21.3)
Unknown	13 (13.8)
Sex partners in the previous year, *n* (%)	
⩽5	32 (34.0)
6–10	12 (12.8)
11–20	14 (14.9)
21–30	2 (2.1)
31–40	6 (6.4)
41–50	4 (4.3)
>50	12 (12.8)
Unknown	12 (12.8)

*N*, number; MSM, men who have sex with men; IDU, injecting drug users.

Gonococcal infections were symptomatic in all 42 patients presenting with urethral infections (Table 2); symptomatic rates for other infection sites were 40% of rectal, 37.5% of cervical and one of 19 patients with pharyngeal infection. Positive samples from asymptomatic patients were predominantly recovered from extragenital sites (70.4%) (*P* *<* 0.01).

**Table 2.** Clinical characteristics of the study population

Clinical characteristics	
Anatomical site, *n* = 104 (%)	
Urethra	42 (40.4)
Rectum	35 (33.7)
Pharynx	19 (18.3)
Cervix	8 (7.7)
Symptomatology, *n* = 104 (%)	
Yes	60 (57.7)
No	43 (41.3)
Unknown	1 (1.0)
Treatment for gonorrhoea, *n* = 95^a^ (%)	
Ceftriaxone^b^ + azithromycin^c^	51 (53.7)
Ceftriaxone^b^	40 (42.1)
Azithromycin^c^	2^d^ (2.1)
No treatment	2 (2.1)
Test of cure, *n* = 104 (%)	
Yes	37 (35.6)
No	67 (64.4)
*Co-infections*	
Gonorrhoea in others sites, *n* = 95^a^ (%)	
Negative	76 (80.0)
Positive	19^e^ (20.0)
Pharyngeal + rectal	9 (9.5)
Cervical + pharyngeal	5 (5.3)
Urethral + rectal	2 (2.1)
Urethral + pharyngeal + rectal	2 (2.1)
Urethral + pharyngeal	1 (1.1)
HIV status, *n* = 94 (%)	
Positive	32 (34.0)
Negative	57 (60.6)
Unknown	5 (5.3)
Other STI different from gonorrhoea, *n* = 95^a^ (%)	
Negative	72 (75.8)
Positive	23 (24.2)
*Chlamydia trachomatis*	12^f^ (12.6)
Rectal	7 (7.4)
Pharyngeal	3 (3.2)
Urethral	2 (2.1)
Cervical	1 (1.1)
LGV	5 (5.3)
Rectal	4 (4.2)
Anal ulcer	1 (1.1)
Condylomas	6 (6.3)
Genital	3 (3.2)
Perianal	3 (3.2)
Primary syphilis	3 (3.2)
Urethritis due to *Ureaplasma urealyticum*	1 (1.1)
Other STI in the previous year, *n* = 94 (%)	
Unknown	1 (1.1)
Negative	59 (62.8)
Positive	34 (36.2)
*Neisseria gonorrhoeae*	23^g^ (24.5)
Rectal	14 (14.9)
Pharyngeal	8 (8.5)
Urethral	6 (6.4)
*Chlamydia trachomatis*	8 (8.5)
Rectal	7 (7.4)
Urethral	1 (1.1)
LGV rectal	1 (1.1)
Syphilis	14 (14.9)
Primary	9 (9.6)
Secondary	3 (3.2)
Early latent syphilis	1 (1.1)
LSUD	1 (1.1)
Condylomas	4 (4.3)
Genital	1 (1.1)
Perianal	3 (3.2)
Urethritis due to *Mycoplasma hominis*	1 (1.1)
Urethritis due to *Ureaplasma urealyticum*	3 (3.2)
Genital herpes (VHS-2)	2 (2.1)
*Molluscum contagiosum*	1 (1.1)
Pediculosis pubis	2 (2.1)

*N*, number; HIV, human immunodeficiency virus; STI, sexually transmitted infections; LGV, *Lymphogranuloma venereum*; LSUD, latent syphilis of unknown duration.

a One patient had two episodes in the period of study.

b Ceftriaxone 250 IM in a single dose.

c Azithromycin 1 g orally in a single dose.

d Two patients received azithromycin as an empirical treatment before learning the aetiology of the infection. Subsequently, they did not go to receive the correct treatment.

e There were 19 patients with *N. gonorrhoeae* infection in several anatomical sites, 12 isolates were not included in this study due to seven strains were only positive by PCR and the remaining five isolates could not be conserved.

f One patient had *C. trachomatis* in two localisations.

g Five patients had *N. gonorrhoeae* infection in several anatomical sites.

Only half of the patients (53.7%) received the recommended dual therapy (ceftriaxone plus azithromycin), while the remaining half were treated only with ceftriaxone (40%) or azithromycin (2%), or did not receive any treatment (2%).

The therapeutic cure rate was 35.6% of cases with a median of 51 days (range: 8–117). Of these, five cases were *N. gonorrhoeae* PCR-positive (median 85 days, range: 50–91), but only in one of them, the strain proved to be of a different ST different from the primary isolate.

Twenty per cent of patients presented with gonococcal infections in more than one body site with rectal and pharyngeal infections being the most frequent combination. Only 28 isolates from these patients could be included in the study; in five cases, the isolates were not stored, and in seven cases, although positives by PCR, an isolate could not be obtained. Thirty-two patients were co-infected with human immunodeficiency virus (HIV), all of them males (*P* = 0.047). Other different HIV and gonorrhoea STIs were also presented in 23 of the total patients, half of them (13) co-infected with *Chlamydia trachomatis* (Table 2).

### Antimicrobial susceptibility

The antimicrobial susceptibility of isolates is summarised in Table 3. All penicillin-resistant isolates (*n* = 16) were positive for *β*-lactamase production. According to CLSI breakpoints, no resistance to ceftriaxone or cefixime was detected. However, three isolates showed cefixime MICs of 0.19 mg/l which is slightly above the EUCAST resistant breakpoint of >0.125 mg/l. Likewise, all isolates were susceptible to spectinomycin but one showed intermediate susceptibility (MIC = 48 mg/l) by CLSI. Only two isolates showed azithromycin resistance (MIC = 1.5 and 2 mg/l), and six were classified as intermediate susceptible (MIC range = 0.38–0.5 mg/l).

**Table 3.** Antimicrobial susceptibility of *Neisseria gonorrhoeae* isolates

Antimicrobial	MIC range (mg/l)	MIC_50_ (mg/l)	MIC_90_ (mg/l)	EUCAST^a^			CLSI^b^		
				S (%)	I (%)	R (%)	S (%)	I (%)	R (%)
Penicillin	0.012–>32	0.25	8	4.8	79.8	^c^15.4	4.8	79.8	15.4^c^
Cefixime	<0.016–0.19	<0.016	0.047	97.1	–	2.9	100	–	–
Ceftriaxone	<0.016–0.094	<0.016	0.047	100	–	–	100	–	–
Azithromycin	0.023–2	0.125	0.25	92.3	5.8	1.9	NA	NA	NA
Ciprofloxacin	<0.002–>32	0.004	16	57.7	–	42.3	57.7	1.9	40.4
Tetracycline	0.032–8	0.38	1	79.8	14.4	5.8	47.1	50.0	2.9
Spectinomycin	2–48	6	12	100	–	–	99	1	–
Gentamicin	0.5–8	3	4	NA	NA	NA	NA	NA	NA

MIC, minimum inhibitory concentration; MIC_50_ and MIC_90_, minimum inhibitory concentration of an antibiotic at which 50% and 90% of the isolates were inhibited, respectively; S, susceptibility; I, intermediate susceptibility; R, resistance; NA, not applicable.

a EUCAST 2016 MIC breakpoints: penicillin: susceptible (*S*) ⩽ 0.06, resistant (*R*) > 1; ceftriaxone: *S* ⩽ 0.125, *R* > 0.125; cefixime: *S* ⩽ 0.125, *R* > 0.125; azithromycin: *S* ⩽ 0.25, *R* > 0.5; ciprofloxacin: *S* ⩽ 0.03, *R* > 0.06; tetracycline: *S* ⩽ 0.5, *R* > 1; spectinomycin: *S* ⩽ 64, *R* > 64.

b CLSI 2016 MIC breakpoints: penicillin: susceptible (*S*) ⩽ 0.06, resistant (*R*) ⩾ 2; ceftriaxone: S ⩽ 0.25; cefixime: *S* ⩽ 0.25; ciprofloxacin: *S* ⩽ 0.06, *R* ⩾ 1; tetracycline: *S* ⩽ 0.25, *R* ⩾ 2; spectinomycin: *S* ⩽ 32, *R* ⩾ 128.

c All penicillin-resistant isolates produced *β*-lactamase.

A high rate of resistance to ciprofloxacin (42.3%/40.4%; EUCAST/CLSI, respectively) was recorded, whereas resistance to tetracycline was relatively uncommon. Gentamicin MICs ranged between 0.5 and 8 mg/l, distributed as a unimodal curve (data not shown); the MIC_50_ and MIC_90_ were 3 and 4 mg/l, respectively. Only five isolates were susceptible to all antimicrobials tested. Overall, most isolates showed resistance to a single antimicrobial, mostly to ciprofloxacin (25.0% and 30.8% EUCAST/CLSI), and generally expressed intermediate susceptibility to penicillin. Eighteen isolates were resistant to two antimicrobials by EUCAST and 11 isolates by CLSI, most of them showing resistance to penicillin and ciprofloxacin (9.6%). Only one isolate was resistant to three antimicrobials (penicillin, tetracycline and ciprofloxacin) according to EUCAST breakpoints. Notably, all cefixime-resistant isolates were also resistant to ciprofloxacin and intermediate resistant to penicillin. Of all antimicrobials tested, only for tetracycline were significant differences evident between EUCAST and CLSI breakpoints criteria (*P* < 0.01).

### Risk factors for antimicrobial resistance and/or decreased susceptibility in MSM.

Since 78 of the 104 isolates originated from MSM, and sexual orientation is highly correlated with many other variables, risk factors analysis was only performed for MSM.

No resistance to ceftriaxone, cefixime or spectinomycin was detected in any of the 78 isolates, and as the prevalence of other AMR in our population was low, the analysis of risk factors was focused on those individuals harbouring isolates clearly resistant to penicillin and ciprofloxacin, and those isolates with DS to tetracycline and azithromycin (Supplementary Tables S1–S3).

Only 15/78 (19.2%) isolates from this patient cohort were fully resistant to penicillin. Nevertheless, univariable logistic regression analysis showed a significant association (*P* < 0.05) between penicillin resistance and the number of sexual partners in the previous year. However, by multivariable analysis, none of the other risk factors (number of sexual partners in the previous year, *P* = 0.04, and HIV status, *P* = 0.08) was significantly associated with penicillin resistance (Table 4). Likewise, none of the foregoing risk factors proved to be significant for individuals harbouring an isolate with DS to penicillin (Table S1).

**Table 4.** Epidemiological and clinical characteristics associated with gonococcal antimicrobial resistance and/or decreased susceptibility by logistic regression analysis, in MSM (*n* = 78)

Characteristics (*n*)	*N* (%)	OR (95% CI)	*P*	aOR (95% CI)	*P*
*Penicillin (R) – EUCAST – CLSI*					
Sex partners in the previous year					
⩽5 (26)	5 (19.2)	1	**0.044**	Excluded	NA
6–10 (12)	1 (8.3)	0.382 (0.040–3.687)		Excluded	
11–20 (10)	4 (40.0)	2.8 (0.567–13.833)		Excluded	
21–30 (2)	2 (100)	0.68E + 10		Excluded	
31–40 (5)	0	NA		Excluded	
41–50 (5)	1 (20.0)	1.050 (0.095–11.558)		Excluded	
>50 (13)	2 (15.4)	0.764 (0.127–4.596)		Excluded	
Unknown (5)	0	NA		Excluded	
*Ciprofloxacin (R) – EUCAST – CLSI*					
Age group					
18–28 (31)	6 (19.4)	1	**0.024**	1	**0.059**
29–39 (24)	13 (54.2)	4.924 (1.484–16.340)		4.607 (1.245–17.044)	
40–51 (23)	9 (39.1)	2.679 (0.789–9.098)		1.436 (0.367–5.613)	
Educational level					
Elementary (3)	3 (100)	0.89E + 10	**0.031**	Excluded	NA
Secondary (13)	2 (15.4)	1		Excluded	
Higher (35)	13 (37.1)	3.250 (0.621–17.013)		Excluded	
Unknown (27)	10 (37.0)	3.235 (0.593–17.658)		Excluded	
IDU					
No (76)	26 (34.2)	1	**0.041**	Excluded	NA
Yes (2)	2 (100)	0.31E + 10		Excluded	
HIV status					
− (40)	7 (17.5)	1	**0**	1	**0.015**
+ (35)	18 (51.4)	4.992 (1.745–14.278)		5.415 (1.733–16.922)	
Unknown (3)	3 (100)	0.76E + 10		0.77E + 10	
Number of anatomical sites with gonorrhoeae					
1 (64)	24 (37.5)	1	**0.042**	Excluded	NA
2 (12)	2 (16.7)	0.333 (0.067–1.652)		Excluded	
3 (2)	2 (100)	0.27E + 10		Excluded	
*Tetracycline (I* *+* *R) – EUCAST*					
Age group					
18–28 (31)	2 (6.5)	1	**0.029**	1	**0.030**
29–39 (24)	8 (33.3)	7.250 (1.371–38.335)		9.196 (1.577–53.639)	
40–51 (23)	3 (13.0)	2.175 (0.333–14.221)		2.161 (0.313–14.939)	
Educational level					
Elementary (3)	3 (100)	0.89E + 10	**0.005**	Excluded	NA
Secondary (13)	2 (15.4)	1		Excluded	
Higher (35)	3 (8.6)	0.516 (0.076–3.502)		Excluded	
Unknown (27)	5 (18.5)	1.250 (0.208–7.505)		Excluded	
Other STI in the previous year					
No (46)	4 (8.7)	1	**0.020**	1	**0.024**
Yes (31)	9 (29.0)	4.295 (1.187–15.539)		5.108 (1.242–21.005)	
Unknown (1)	0	NA		NA	
Number of anatomical sites with gonorrhoeae					
1 (64)	11 (17.2)	1	**0.003**	Excluded	NA
2 (12)	0	NA		Excluded	
3 (2)	2 (100)	0.78E + 10		Excluded	
*Tetracycline (I* *+* *R) – CLSI*					
Educational level					
Elementary (3)	3 (100)	0.05E + 10	**0.009**	0.01E + 10	**0.031**
Secondary (13)	10 (76.9)	1		1	
Higher (35)	12 (34.3)	0.323 (0.072–1.440)		0.071 (0.012–0.405)	
Unknown (27)	14 (51.9)	0.157 (0.036–0.679)		0.158 (0.028–0.897)	
Other STI in the previous year					
No (46)	19 (41.3)	1	**0.045**	1	**0.004**
Yes (31)	20 (64.5)	2.584 (1.008–6.622)		6.243 (1.797–21.686)	
Unknown (1)	0	NA		NA	
Number of anatomical sites with gonorrhoeae					
1 (64)	26 (40.63)	1	**0.001**	1	**0.016**
2 (12)	11 (91.7)	16.077 (1.955–132.211)		27.589 (2.865–265.686)	
3 (2)	2 (100)	0.24E + 10		6.243	

*n*, number of *N. gonorrhoeae* strains isolated from MSM; MIC, minimum inhibitory concentration; Penicillin (R) – EUCAST – CLSI, penicillin resistance (MIC ⩾ 1 mg/l) according to EUCAST and CLSI breakpoints; Ciprofloxacin (R) – EUCAST – CLSI, ciprofloxacin resistance (MIC > 0.06 mg/l) according to EUCAST and CLSI breakpoints; Tetracycline (I + R) – EUCAST, tetracycline decreased susceptibility (MIC > 0.5 mg/l) according to EUCAST breakpoints; Tetracycline (I + R) – CLSI, tetracycline decreased susceptibility (MIC > 0.25 mg/l) according to CLSI breakpoints; *N*, number of penicillin-resistant strains/ciprofloxacin-resistant strains/tetracycline decreased susceptible isolates; OR, odds ratio; CI, confident interval; aOR, adjusted OR; NA, not applicable; Excluded, variable excluded by backward method in logistic regression analysis. *P* < 0.05 are shown in bold.

By univariate analysis, significant associations (*P* < 0.05) with age, educational level, history of intravenous drug use, HIV status and number of gonococcal-infected sites were observed for MSM with ciprofloxacin-resistant isolates (28/78). However, only HIV-positive patients remained significantly associated with such resistance on multivariable regression analysis (adjusted odds ratio (aOR) = 5.415; 95% confidence interval (CI) 1.733–16.922; *P* = 0.015) (Table 4, Table S2).

Of the 78 MSM isolates, only three and two (EUCAST/CLSI breakpoints, respectively) showed tetracycline resistance; and 10 (12.8%) and 37 (47.4%) isolates exhibited DS to this agent. Educational level, other STI in the previous year and number of gonococcal-infected sites were independent factors significantly associated (*P* < 0.05) with the latter. Notably, age was also significantly associated with EUCAST breakpoint alone; tetracycline decreased susceptible isolates, by both breakpoint criteria, were significantly associated in the adjusted analysis with having had an STI in the previous year (aOR = 5.108/6.243; 95% CI 1.242–21.005/1.797–21.686; *P* = 0.024/0.004 EUCAST/CLSI). The 29–39 age group was also significantly associated with tetracycline DS (EUCAST aOR = 9.196; 95% CI 1.577–53.639; *P* = 0.03); and two gonorrhoea-infected sites with CLSI tetracycline (aOR = 27.589; 95%CI 2.865–265.686; *P* = 0.016) (Table 4, Table S3).

Age was the only independent factor significantly associated with azithromycin decreased susceptible isolates (*P* = 0.047) (Table S2).

### Serotyping and molecular characterisation

**Fig. 1.**
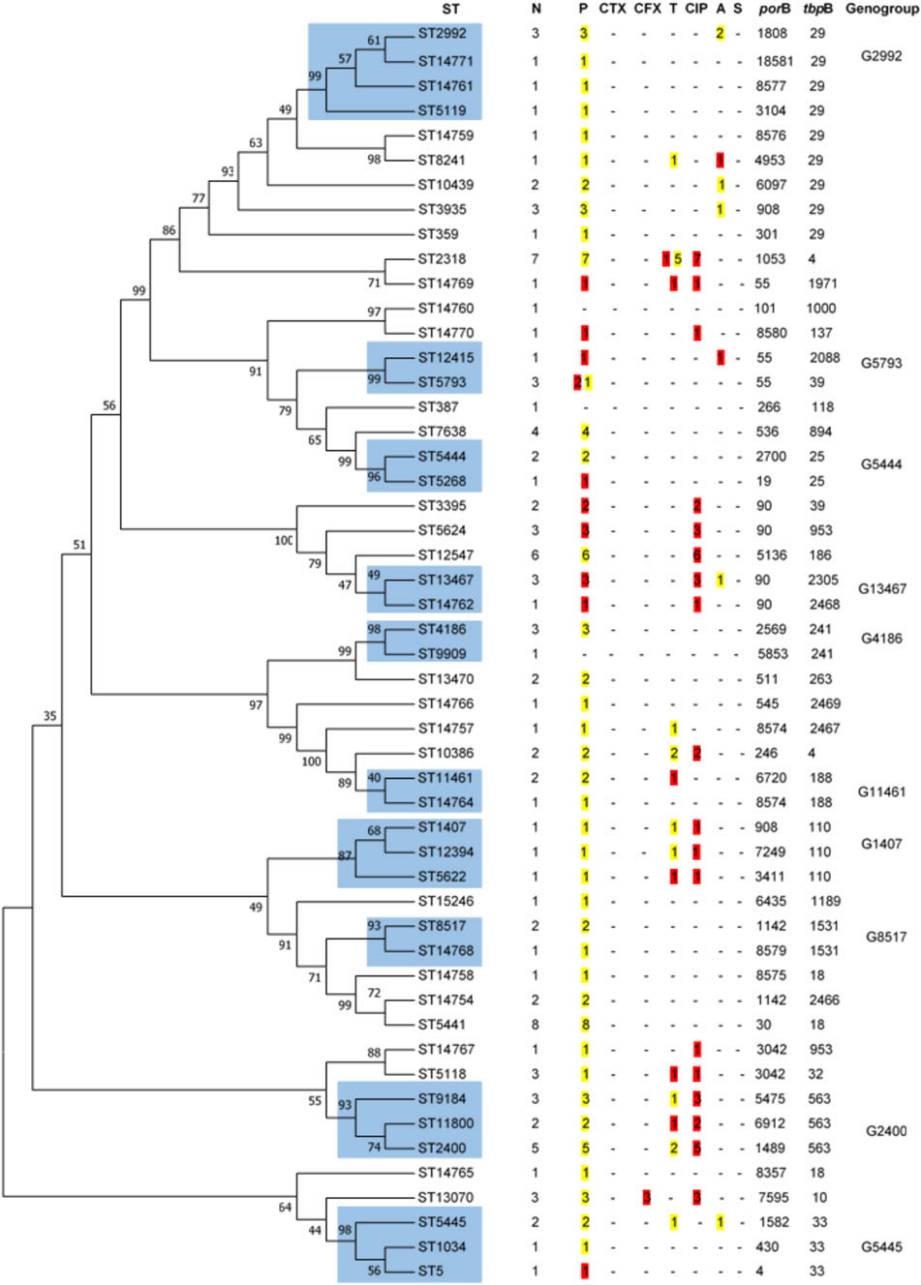
Dendogram constructed by Neighbor-Joining method applying 1000 replicates of bootstrap. The similarities between the 51 STs of *N. gonorrhoeae* strains were analysed using the maximum likelihood test after concatenating *porB* and *tbpB* sequences. In blue are shown the cluster of STs that share 99% of identity and belong to the same genogroup. N, number. P, penicillin. CTX, ceftriaxone. CFX, cefixime. T, tetracycline. CIP, ciprofloxacin. A, azithromycin. S, spectinomycin. In yellow are shown the number of isolates with intermediate susceptibility and in red with resistance following EUCAST breakpoints.

All isolates were serotype IB. Although 51 different STs were found by NG-MAST (Fig. 1), representing 42 and 28 different *porB* and *tbpB* alleles, most of the isolates (74.0%) fell into 24 STs; the remaining 27 STs each representing a single strain. The most predominant STs were ST5441 (*n* = 8), ST2318 (*n* = 7), ST12547 (*n* = 6), ST2400 (*n* = 5) and ST7638 (*n* = 4). Sixteen STs (ST14754, ST14757, ST14758, ST14759, ST14760, ST14761, ST14762, ST14764, ST14765, ST14766, ST14767, ST14768, ST14769, ST14770, ST14771 and ST15246), seven *por*B alleles (8574, 8575, 8576, 8577, 8579, 8580 and 8581) and four *tbp*B alleles (2466, 2467, 2468 and 2469) had not been previously described.

Closely related STs were clustered into 10 genogroups (Fig. 1) encompassing 44 of the 104 isolates; the most frequent were G2400 (three STs, ten strains) followed by G2992 (four STs, six strains). There were four genogroups each comprising four isolates, G4186 (two STs), G5445 (three STs), G5793 (two STs) and G13467 (two STs). The less prevalent genogroups, each with three isolates, were G1407 (three STs), G5444 (two STs), G8517 (two STs) and G11461 (two STs).

STs 5441 and 14754 do not meet the requirements to be classified as a genogroup due to the fact that do not share either *por*B or *tbp*B alleles. However, these STs differ by <1%, i.e. only 2 bp in *por*B alleles and 1 bp in *tbp*B alleles.

Cefixime-resistant strains belonged to ST13070, and the azithromycin-resistant strains were characterised as ST8241 and ST12415. The single ST1407 strain was resistant to ciprofloxacin, showed DS to penicillin and tetracycline, but was ceftriaxone and cefixime susceptible (MICs 0.094 and 0.047 mg/l, respectively).

The analysis of the more prevalent genogroups and STs, accounting for approximately one-third of the isolates and individual patients, is summarised in Table 5. There were no differences in gender, origin, anatomical site and age among the patients harbouring the more prevalent genogroups and STs, but G2400 was significantly associated with heterosexual subjects (*P* < 0.01). In this cohort, significant differences were found for ciprofloxacin resistance in ST2318 (*P* = 0.031), G2400 (*P* < 0.01), G2992 (*P* < 0.01) and G5441 (*P* < 0.01); for tetracycline in ST2318 (*P* < 0.01) and for azithromycin in G2992 (*P* = 0.023).

**Table 5.** Epidemiological and clinical characteristics of the more prevalent genogroups and STs

	G2400 (*n* = 10)	ST5441 (*n* = 8)	ST2318 (*n* = 7)	ST12547 (*n* = 6)	G2992 (*n* = 6)
NG-MAST ST (*n*)	ST2400 (*n* = 5) ST9184 (*n* = 3) ST11800 (*n* = 2)	ST5441 (*n* = 8)	ST2318 (*n* = 7)	ST12547 (*n* = 6)	ST2992 (*n* = 3) ST5119 (*n* = 1) ST14761 (*n* = 1) ST14771 (*n* = 1)
Penicillin	Intermediate	Intermediate	Intermediate	Intermediate	Intermediate
Ceftriaxone	Susceptibility	Susceptibility	Susceptibility	Susceptibility	Susceptibility
Cefixime	Susceptibility	Susceptibility	Susceptibility	Susceptibility	Susceptibility
Tetracycline	Variable^a^	Susceptibility	**Variable^b^**	Susceptibility	Susceptibility
Ciprofloxacin	**Resistance**	**Susceptibility**	**Resistance**	Resistance	**Susceptibility**
Azithromycin	Susceptibility	Susceptibility	Susceptibility	Susceptibility	**Variable^c^**
Spectinomycin	Susceptibility	Susceptibility	Susceptibility	Susceptibility	Susceptibility
Genital infection (%)	70	62.5	28.6	83.3	50
Subjects (*n*)	8	8	6	6	5
Median age, years (range)	33.5 (22–46)	35.5 (19–50)	32.5 (19–49)	37.5 (22–44)	38 (23–49)
Males (%)	75	100	100	100	80
Homosexual (%)	25	87.5	83.3	83.3	60
European (%)	75	62.5	33.3	83.3	60
HIV+ (%)	0	25	50	50	20

*N*, number. Antimicrobial susceptibility values were interpreted in accordance with EUCAST. Parameters in which significant differences were found are shown in bold.

a Variable: 60% (*n* = 6) susceptibility, 30% (*n* = 3) intermediate and 10% (*n* = 1) resistance.

b Variable: 14.3% (*n* = 1) susceptibility, 71.4% (*n* = 5) intermediate and 14.3% (*n* = 1) resistance.

c Variable: 66.7% (*n* = 4) susceptibility and 33.3% (*n* = 2) intermediate.

## Discussion

A randomly selected sample of 104 *N. gonorrhoeae* isolates recovered from patients attending an STI outpatient clinic in Madrid over a 4-month period in 2016 was investigated to determine their antimicrobial susceptibility and molecular epidemiology, and explore statistical associations of isolate characteristics with socio-demographic, behavioural and clinical factors of patients.

We detected resistance to penicillin (15.4%), ciprofloxacin (42.3%/40.4% EUCAST/CLSI criteria), tetracycline (5.8%/2.9% EUCAST/CLSI), cefixime (2.5% EUCAST) and azithromycin (1.9%). According to the 2015 European Gonococcal Antimicrobial Surveillance Programme (Euro-GASP) [8], the observed rates were similar to the European average, except for azithromycin (7.1%), and to the overall average for Spain, except for ciprofloxacin (65.3%). Contrary to our study, most of the Spanish strains included in 2015 Euro-GASP were isolated from heterosexual men (21.3% *vs.* 60%) in which the highest ciprofloxacin resistance rate was found [8]. A significant association between heterosexual men and ciprofloxacin resistance has been previously described in other Spanish regions [22, 23], and this was confirmed in the present study with ciprofloxacin resistance being highest in heterosexual males (70%), but was half as frequent in MSM.

In our study, resistance to azithromycin was rare (1.9%), well below the previously observed European average in 2015 (7.1%), and lower than previously reported from Spain for 2009–2014 [8, 23, 24]. Azithromycin resistance in Spain averaged 3% in 2015, and showed a continued decline compared to previous years [8], which was also found in our study sample.

If CLSI criteria are applied, all isolates tested were susceptible to cefixime and ceftriaxone, whereas according to EUCAST criteria, three strains (2.9%) were cefixime-resistant (MIC = 0.19 mg/l). Comparing with other Spanish regions, Serra-Pladevall *et al*. [25] reported a cefixime resistance rate of 8.2% among gonococcal isolates during 2013 in Barcelona and 0.6% resistance to ceftriaxone. Likewise, Cobo *et al*. found the rates of cefixime and ceftriaxone resistance were 6.1% and 4.6%, respectively, in 65 isolates collected during 2012–2014 in southern Spain [24]. In this sense, as is observed in our study, the resistance to extended spectrum cephalosporins appears to have been decreasing in recent years. Indeed, the 2015 Euro-GASP report [8] gave an average of 2.4% in Spain with no resistance to ceftriaxone.

Even though there remain some differences between EUCAST and CLSI criteria [17, 18], we only found significant differences between tetracycline resistance rates (*P* < 0.01), thus confirming efforts to harmonise the clinical breakpoints.

European guidelines recommend the use of dual therapy with ceftriaxone and azithromycin for uncomplicated gonorrhoea [5] in order to improve therapeutic efficacy and slow the emergence of resistance to cephalosporins. Moreover, the use of dual therapy is justified by the high number of people co-infected with gonorrhoea and chlamydia [13], although rates of azithromycin resistance above 5% of threshold in *C. trachomatis* and *Mycoplasma genitalium* infections have been observed in some studies [26–28]. According to our data, neither cephalosporins nor azithromycin reaches a resistance threshold of 5% in our specific population, so, perhaps dual therapy should not be recommended for uncomplicated gonorrhoea infections in order to prevent the emergence of increased resistance to azithromycin in other bacterial STIs.

Most of the patients (95.7%) were administered ceftriaxone with or without azithromycin as treatment, two patients were treated only with azithromycin and treatment was unknown for the remaining two. Clinical cure was achieved in over a third of patients although five cases again became culture-positive; however, in the latter cases, test of cure was performed over a range of 60–120 days, so these most likely represent reinfections rather than treatment failures. Test of cure is recommended in all gonorrhoea cases to ensure effective eradication, especially for pharyngeal infection which is substantially harder to treat than genital and rectal infections, and identify emerging resistance [5, 6].

Some guidelines recommend the use of gentamicin when treatment failures with cephalosporins are suspected [5, 6]. Gentamicin was first introduced for syndromic management of urethritis in Malawi in 1993, and there has been no reported increase in the number of therapeutic failures [29]. Moreover, a clinical trial showed that gentamicin was no clinically worse than ceftriaxone for gonorrhoea treatment [30] and a microbiological cure was achieved for all patients who received gentamicin 240 mg intramuscularly plus azithromycin 2 g orally [31]. Although there are no official clinical breakpoints for gentamicin, high rates of *in vitro* susceptibility have been shown in Europe [32]. According to the Malawi established breakpoints [33], none of the isolates studied here showed gentamicin resistance (MIC ⩾ 32 mg/l); the great majority (95.2%) were gentamicin susceptible (MIC ⩽ 4 mg/l), and only five isolates were classed as intermediate susceptible (MIC = 6 mg/l, 4; and MIC = 8 mg/l).

As in other countries such as Germany and the UK, no spectinomycin-resistant isolates [34, 35] were found. This antimicrobial is not available in many countries so its limited use might explain the lack of resistance. Overall, with the exception of ciprofloxacin, relatively low AMR was evident in our population. Nevertheless, continuous antimicrobial surveillance is necessary to monitor the prevalence and distribution of changes in resistance in order to provide quality data for appropriate local treatment guidelines.

Despite the genetic diversity (51 STs) of *N. gonorrhoeae* strains in our study population, just over half of them represented single strains. Most (74%) fell in to 24 different STs, which were clustered in 10 genogroups, with G2400 and G2992 being the most predominant. Contrary to our results, a statistically significant relationship between the latter genogroups and MSM has been previously reported [20, 22]. Although three-quarters of our isolates were recovered from MSM, we found a statistically significant relationship between G2400 and heterosexual patients, while G2992 seemed arbitrarily distributed between MSM and heterosexual subjects. It is also noteworthy that G1407, the most prevalent genogroup seen in the previous studies [20, 22, 24], was one of the less prevalent genogroups found here and represented only by three strains. G1407, which is widely disseminated, has been associated with cefixime and ceftriaxone resistance [3, 20, 36], and specifically with cephalosporin treatment failures internationally [3]. An increase in the testing of extragenital samples combined with appropriate dual therapy (ceftriaxone + azithromycin) may have contributed to reducing the G1407 reservoir in our region. Despite the relatively small size of our study dataset which might not be representative of the general STI population, our data do show a difference to previously reported epidemiology in that the four most prevalent clones (G2400, ST5441, ST2318 and ST12547) were poorly represented in the last Euro-GASP survey at both a European and Spanish level [20]. We observed a decrease in the prevalence of G1407, and an increase in ST5441 and ST2318. Since 71.4% and 37.5% of the ST2318 and ST5441 strains, respectively, were isolated from extragenital samples, these STs could have occupied the G1407 ecological niche. These findings emphasise the need for continuous monitoring of gonococcal strain populations to identify temporal changes and expansion of new clones.

Identification of gonococcal transmission networks, as well as those individuals with a higher risk for acquiring AMR *N. gonorrhoeae*, is needed to focus on treatments and underpin public health intervention strategies. Our study sample was mainly derived from young people (18–28 years) and who were MSM. It is also notable that one-third of the subjects had a higher level of education. In accordance with the previous studies [8, 23–25, 37], in which MSM is generally identified as an increased vulnerable population for both STI and HIV infections, the highest gonococcal positivity rate was for males (89.4%), and particularly from MSM (75.5%). Moreover, significant proportions of our MSM patients were HIV-positive (45.1%), also presented with STI other than gonorrhoea (32.4%), and had had another STI in the previous year (40.8%). Since MSM were predominant, and sexual orientation is highly correlated with many other variables, we analysed the risk factors for AMR and decreased susceptible isolates from MSM. There was a significant association between HIV-positive MSM and carriage of a ciprofloxacin-resistant gonococcal strain. In a previous study in the Netherlands [38], it was observed that infection with *N. gonorrhoeae* resistant to ciprofloxacin was more likely in HIV-positive men than in HIV-negative men, although this was not confirmed by multivariable analysis. However, they did not specifically analyse the MSM population, as performed here. We also found a significant association between age and being carriers of decreased susceptible isolates to azithromycin in MSM, in agreement with the previous studies reporting this significant association with any AMR strain [19, 37, 39].

The relatively low number of patients in this cohort, which was conducted at one STI clinic, and the over-representation of MSM, are the main limitations of our study, and thus may not be a representative sample of the general population.

In conclusion, we found a decrease in the rate of AMR gonococcal isolates which contrasts with some previously reported studies and confirmed the validity of both EUCAST and CLSI breakpoints for the classification of resistance phenotypes. The widely disseminated multi-drug resistant genogroup, G1407, was quite rare, while more susceptible clones such as ST5441, associated with susceptibility to all antimicrobials tested, and ST2318, associated with extragenital locations, were the most prevalent. Continuous molecular and antimicrobial surveillance programmes are necessary to detect population changes and to provide quality data to support treatment guidelines.

## Supplementary material

For supplementary material accompanying this paper visit https://doi.org/10.1017/S095026881900150X. click here to view supplementary material
