# Supplementary material for: Epidemiology, molecular characterisation and antimicrobial susceptibility of Neisseria gonorrhoeae isolates in Madrid, Spain, in 2016
Source: Epidemiol Infect. 2019 Sep 24;147:e274. doi: 10.1017/S095026881900150X (PMC6805738; doi:10.1017/S095026881900150X)
Supplement: Supplementary file 1 [file S095026881900150Xsup001.doc]

Table S1. Epidemiological and clinical characteristics association with gonococcal penicillin resistance (MIC ≥1mg/L) and penicillin decreased susceptibility (MIC >0.06mg/L), by logistic regression analysis, in MSM (n=78).

| Characteristics (n) | **Penicillin (R) - EUCAST - CLSI** | | | | | **Penicillin (I+R) – EUCAST - CLSI** | | |
| --- | --- | --- | --- | --- | --- | --- | --- | --- |
|  | N (%) | OR (95% CI) | P | aOR (95% CI) | P | N (%) | OR (95% CI) | P |
| **Age group** | | | | | | | | |
| 18-28 (31) | 4 (12.9) | 1.00 | 0.317 | NA | NA | 30 (96.8) | 1 | 0.696 |
| 29-39 (24) | 7 (29.2) | 2.779 (0.706-10.940) |  | NA |  | 22 (91.7) | 0.367 (0.031-4.303) |  |
| 40-51 (23) | 4 (17.4) | 1.421 (0.315-6.401) |  | NA |  | 22 (95.7) | 0.733 (0.043-12.376) |  |
| **Origin** | | | | | | | | |
| European (55) | 12 (22.6) | 0.558 (0.047-6.693) | 0.515 | NA | NA | 51 (92.7) | 0 | 0.410 |
| Latin American (18) | 2 (11.1) | 0.250 (0.015-4.172) |  | NA |  | 18 (100) | 0.01E+10 |  |
| Asian (3) | 1 (33.3) | 1.00 |  | NA |  | 3 (100) | 0.01E+10 |  |
| American (2) | 0 | NA |  | NA |  | 2 (100) | 0.01E+10 |  |
| **Educational level** | | | | | | | | |
| Elementary (3) | 0 | NA | 0.448 | NA | NA | 3 (100) | 0.01E+10 | 0.762 |
| Secondary (13) | 2 (15.4) | 1.00 |  | NA |  | 12 (92.3) | 1 |  |
| Higher (35) | 9 (25.7) | 1.904 (0.353-10.283) |  | NA |  | 34 (97.1) | 2.833 (0.164-48.926) |  |
| Unknown (27) | 4 (14.8) | 0.957 (0.151-6.042) |  | NA |  | 25 (92.6) | 1.042 (0.086-12.655) |  |
| **IDU** | | | | | | | | |
| No (76) | 14 (18.4) | 1.00 | 0.321 | NA | NA | 72 (94.7) | 1 | 0.644 |
| Yes (2) | 1 (50.0) | 4.429 (0.261-75.182) |  | NA |  | 2 (100) | 0.01E+10 |  |
| **Recreational drug users** | | | | | | | | |
| No (18) | 1 (5.6) | 1 | 0.163 | NA | NA | 16 (88.9) | 1 | 0.389 |
| Yes (54) | 13 (24.1) | 5.390 (0.653-44.508) |  | NA |  | 52 (92.6) | 3.25 (0.423-24.957) |  |
| Unknown (6) | 1 (16.7) | 3.400 (0.179-64.682) |  | NA |  | 6 (100) | 0.02E+10 |  |
| **Sex partners in previous year** | | | | | | | | |
| <=5 (26) | 5 (19.2) | 1 | 0.044 | Excluded | NA | 25 (96.2) | 1 | 0.568 |
| 6-10 (12) | 1 (8.3) | 0.382 (0.040-3.687) |  | Excluded |  | 10 (83.3) | 0.200 (0.016-2.461) |  |
| 11-20 (10) | 4 (40.0) | 2.8 (0.567-13.833) |  | Excluded |  | 9 (90) | 0.360 (0.020-6.379) |  |
| 21-30 (2) | 2 (100) | 0.68E+10 |  | Excluded |  | 2 (100) | 0.01E+10 |  |
| 31-40 (5) | 0 | NA |  | Excluded |  | 5 (100) | 0.01E+10 |  |
| 41-50 (5) | 1 (20.0) | 1.050 (0.095-11.558) |  | Excluded |  | 5 (100) | 0.01E+10 |  |
| >50 (13) | 2 (15.4) | 0.764 (0.127-4.596) |  | Excluded |  | 13 (100) | 0.01E+10 |  |
| Unknown (5) | 0 | NA |  | Excluded |  | 5 (100) | 0.01E+10 |  |
| **Anatomical localization** | | | | | | | | |
| Urethra (30) | 8 (26.7) | 1 | 0.293 | NA | NA | 29 (96.7) | 1 | 0.818 |
| Rectum (35) | 6 (17.1) | 0.569 (0.172-1.879) |  | NA |  | 33 (94.3) | 0.569 (0.049-6.605) |  |
| Pharynx (13) | 1 (7.7) | 0.229 (0.026-2.057) |  | NA |  | 12 (92.3) | 0.414 (0.024-7.170) |  |
| **Symptomatology** | | | | | | | | |
| No (32) | 4 (12.5) | 1 | 0.183 | NA | NA | 30 (93.75) | 1 | 0.892 |
| Yes (45) | 11 (24.4) | 2.265 (0.650-7.896) |  | NA |  | 43 (95.6) | 1.433 (0.191-10.747) |  |
| Unknown (1) | 0 | NA |  | NA |  | 1 (100) | 0.01E+10 |  |
| **HIV status** | | | | | | | | |
| - (40) | 5 (12.5) | 1 | 0.081 | 1 | 0.090 | 36 (90) | 1 | 0.062 |
| + (35) | 10 (28.6) | 2.800 (0.852-9.203) |  | 2.800 (0.852-9.203) |  | 35 (100) | 0.02E+10 |  |
| Unknown (3) | 0 | NA |  | NA |  | 3 (100) | 0.02E+10 |  |
| **Other STI different from Gonorrhoea** | | | | | | | | |
| No (54) | 12 (22.2) | 1 | 0.299 | NA | NA | 51 (94.4) | 1 | 0.794 |
| Yes (24) | 3 (12.5) | 0.500 (0.127-1.966) |  | NA |  | 23 (95.8) | 1.353 (0.133-13.713) |  |
| Other STI in the previous year | | | | | | | | |
| No (46) | 8 (17.4) | 1 | 0.575 | NA | NA | 43 (93.5) | 1 | 0.764 |
| Yes (31) | 7 (22.6) | 1.385 (0.445-4.314) |  | NA |  | 30 (96.8) | 2.093 (0.208-21.101) |  |
| Unknown (1) | 0 | NA |  | NA |  | 1 (100) | 0.01E+10 |  |
| **Number of anatomical sites with gonorrhoea** | | | | | | | | |
| 1 (64) | 15 (23.1) | NA | NA | NA | NA | 60 (93.75) | 1 | 0.443 |
| 2 (12) | 0 | NA |  | NA |  | 12 (100) | 0.01E+10 |  |
| 3 (2) | 0 | NA |  | NA |  | 2 (100) | 0.01E+10 |  |

n: number of *N. gonorrhoeae* strains isolated from MSM.

MIC: minimum inhibitory concentration.

Penicillin (R) - EUCAST - CLSI: Penicillin resistance (MIC ≥1mg/L) according to EUCAST and CLSI breakpoints.

Penicillin (I+R) - EUCAST - CLSI: Penicillin decreased susceptibility (MIC >0.06mg/L) according to EUCAST and CLSI breakpoints.

N: number of penicillin resistant or decreased susceptible isolates.

OR: odds ratio.

CI: confident interval.

aOR: adjusted OR.

NA: not applicable.

Excluded: variable excluded by backward method in logistic regression analysis.

Table S2. Epidemiological and clinical characteristics association with gonococcal ciprofloxacin resistance (MIC >0.06mg/L) and azithromycin decreased susceptibility (MIC ≥0.5mg/L), by logistic regression analysis, in MSM (n=78).

| Characteristics | **Ciprofloxacin (R) – EUCAST - CLSI** | | | | | **Azithromycin (I+R) – EUCAST** | | | | |
| --- | --- | --- | --- | --- | --- | --- | --- | --- | --- | --- |
|  | N (%) | OR (95% CI) | P | aOR (95% CI) | P | N (%) | OR (95% CI) | P | aOR (95% CI) | P |
| **Age group** | | | | | | | | | | |
| 18-28 (31) | 6 (19.4) | 1 | 0.024 | 1 | 0.059 | 5 (16.1) | 1 | 0.047 | Excluded | NA |
| 29-39 (24) | 13 (54.2) | 4.924 (1.484-16.340) |  | 4.607 (1.245-17.044) |  | 0 | NA |  | Excluded |  |
| 40-51 (23) | 9 (39.1) | 2.679 (0.789-9.098) |  | 1.436 (0.367-5.613) |  | 2 (8.7) | 0.495 (0.087-2.815) |  | Excluded |  |
| **Origin** | | | | | | | | |  |  |
| European (55) | 18 (32.7) | 1 | 0.808 | NA | NA | 5 (9.1) | 1 | 0.562 | NA | NA |
| Latin American (18) | 8 (44.4) | 1.644 (0.555-4.876) |  | NA |  | 1 (5.6) | 0.588 (0.064-5.397) |  | NA |  |
| Asian (3) | 1 (33.3) | 1.028 (0.087-12.098) |  | NA |  | 1 (33.3) | 5 (0.382-65.360) |  | NA |  |
| American (2) | 1 (50) | 2.056 (0.121-34.779) |  | NA |  | 0 | NA |  | NA |  |
| **Educational level** | | | | | | | | | | |
| Elementary (3) | 3 (100) | 0.89E+10 | 0.031 | Excluded | NA | 0 | NA | 0.474 | NA | NA |
| Secondary (13) | 2 (15.4) | 1 |  | Excluded |  | 2 (15.4) | 1 |  | NA |  |
| Higher (35) | 13 (37.1) | 3.250 (0.621-17.013) |  | Excluded |  | 4 (11.4) | 0.710 (0.114-4.431) |  | NA |  |
| Unknown (27) | 10 (37.0) | 3.235 (0.593-17.658) |  | Excluded |  | 1 (3.7) | 0.212 (0.017-2.582) |  | NA |  |
| **IDU** | | | | | | | | | | |
| No (76) | 26 (34.2) | 1 | 0.041 | Excluded | NA | 7 (9.2) | 1 | 0.537 | NA | NA |
| Yes (2) | 2 (100) | 0.31E+10 |  | Excluded |  | 0 | NA |  | NA |  |
| **Recreational drug users** | | | | | | | | |  |  |
| No (18) | 5 (27.8) | 1 | 0.683 | NA | NA | 2 (11.1) | 1 | 0.541 | NA | NA |
| Yes (54) | 21 (38.9) | 1.655 (0.515-5.318) |  | NA |  | 5 (9.3) | 0.816 (0.144-4.624) |  | NA |  |
| Unknown (6) | 2 (33.3) | 1.3 (0.178-9.474) |  | NA |  | 0 | NA |  | NA |  |
| **Sex partners in previous year** | | | | | | | | | | |
| <=5 (26) | 7 (26.9) | 1 | 0.069 | Excluded | NA | 3 (11.5) | 1 | 0.236 | NA | NA |
| 6-10 (12) | 4 (33.3) | 1.357 (0.309-5.964) |  | Excluded |  | 0 | NA |  | NA |  |
| 11-20 (10) | 6 (60.0) | 4.071 (0.879-18.868) |  | Excluded |  | 3 (30.0) | 3.286 (0.538-20.081) |  | NA |  |
| 21-30 (2) | 2 (100) | 0.44E+10 |  | Excluded |  | 0 | NA |  | NA |  |
| 31-40 (5) | 1 (20.0) | 0.679 (0.064-7.161) |  | Excluded |  | 0 | NA |  | NA |  |
| 41-50 (5) | 2 (40.0) | 1.810 (0.248-13.211) |  | Excluded |  | 0 | NA |  | NA |  |
| >50 (13) | 6 (46.2) | 2.327 (0.578-9.367) |  | Excluded |  | 1 (7.7) | 0.639 (0.060-6.823) |  | NA |  |
| Unknown (5) | 0 | NA |  | Excluded |  | 0 | NA |  | NA |  |
| **Anatomical localization** | | | | | | | | | | |
| Urethra (30) | 13 (43.3) | 1 | 0.559 | NA | NA | 2 (6.7) | 1 | 0.787 | NA | NA |
| Rectum (35) | 11 (31.4) | 0.599 (0.217-1.654) |  | NA |  | 4 (11.4) | 1.806 (0.307-10.633) |  | NA |  |
| Pharynx (13) | 4 (30.8) | 0.581 (0.146-2.314) |  | NA |  | 1 (7.7) | 1.167 (0.096-14.126) |  | NA |  |
| **Symptomatology** | | | | | | | | | | |
| No (32) | 11 (34.4) | 1 | 0.609 | NA | NA | 3 (93.8) | 1 | 0.907 | NA | NA |
| Yes (45) | 17 (37.8) | 1.159 (0.450-2.986) |  | NA |  | 4 (8.9) | 0.943 (0.196-4.536) |  | NA |  |
| Unknown (1) | 0 | NA |  | NA |  | 0 | NA |  | NA |  |
| **HIV status** | | | | | | | | | | |
| - (40) | 7 (17.5) | 1 | 0 | 1 | 0.015 | 3 (7.5) | 1 | 0.560 | NA | NA |
| + (35) | 18 (51.4) | 4.992 (1.745-14.278) |  | 5.415 (1.733-16.922) |  | 4 (11.4) | 1.591 (0.331-7.658) |  | NA |  |
| Unknown (3) | 3 (100) | 0.76E+10 |  | 0.77E+10 |  | 0 | NA |  | NA |  |
| **Other STI different from Gonorrhoea** | | | | | | | | | | |
| No (54) | 17 (31.5) | 1 | 0.226 | NA | NA | 5 (9.3) | 1 | 0.894 | NA | NA |
| Yes (24) | 11 (45.8) | 1.842 (0.686-4.942) |  | NA |  | 2 (8.3) | 0.891 (0.160-4.952) |  | NA |  |
| Other STI in the previous year | | | | | | | | | | |
| No (46) | 17 (37.0) | 1 | 0.633 | NA | NA | 5 (10.9) | 1 | 0.725 | NA | NA |
| Yes (31) | 11 (35.5) | 0.938 (0.363-2.422) |  | NA |  | 2 (6.5) | 0,566 (0.103-3.119) |  | NA |  |
| Unknown (1) | 0 | NA |  | NA |  | 0 | NA |  | NA |  |
| **Number of anatomical sites with gonorrhoeae** | | | | | | | | | | |
| 1 (64) | 24 (37.5) | 1 | 0.042 | Excluded | NA | 4 (6.3) |  | 0.067 | 1 | 0.054 |
| 2 (12) | 2 (16.7) | 0.333 (0.067-1.652) |  | Excluded |  | 3 (25) | 5.000 (0.958-26.108) |  | 5.083 (0.974-26.534) |  |
| 3 (2) | 2 (100) | 0.27E+10 |  | Excluded |  | 0 | NA |  | NA |  |

n: number of *N. gonorrhoeae* strains isolated from MSM.

MIC: minimum inhibitory concentration.

Ciprofloxacin (R) - EUCAST - CLSI: Ciprofloxacin resistance (MIC > 0.06 mg/L) according to EUCAST and CLSI breakpoints.

Azithromycin (I+R) - CLSI: Azithromycin decreased susceptibility (MIC ≥ 0.5 mg/L) according to CLSI breakpoints.

N: number of ciprofloxacin resistant strains / azithromycin decreased susceptible isolates.

OR: odds ratio.

CI: confident interval.

aOR: adjusted OR.

NA: not applicable.

Excluded: variable excluded by backward method in logistic regression analysis.

Table S3. Epidemiological and clinical characteristics association with gonococcal tetracycline decreased susceptibility (MIC > 0.5mg/L and MIC > 0.25mg/l according to EUCAST and CLSI breakpoints, respectively), by logistic regression analysis, in MSM (n=78).

| Characteristics (n) | **Tetracycline (I+R) - EUCAST** | | | | | **Tetracycline (I+R) – CLSI** | | | | |
| --- | --- | --- | --- | --- | --- | --- | --- | --- | --- | --- |
|  | N (%) | OR (95% CI) | P | aOR (95% CI) | P | N (%) | OR (95% CI) | P | aOR (95% CI) | P |
| **Age group** | | | | | | | | | | |
| 18-28 (31) | 2 (6.5) | 1 | 0.029 | 1 | 0.030 | 14 (45.2) | 1 | 0.778 | NA | NA |
| 29-39 (24) | 8 (33.3) | 7.250 (1.371-38.335) |  | 9.196 (1.577-53.639) |  | 13 (54.2) | 1.435 (0.492-4.184) |  | NA |  |
| 40-51 (23) | 3 (13.0) | 2.175 (0.333-14.221) |  | 2.161 (0.313-14.939) |  | 15 (52.2) | 1.325 (0.449-3.907) |  | NA |  |
| **Origin** | | | | | | | | |  |  |
| European (55) | 8 (14.5) | 1 | 0.335 | NA | NA | 29 (52.7) | 1 | 0.062 | Excluded | NA |
| Latin American (18) | 5 (27.8) | 2.260 (0.631-8.087) |  | NA |  | 10 (55.6) | 1.121 (0.384-3.267) |  | Excluded |  |
| Asian (3) | 0 | NA |  | NA |  | 0 | NA |  | Excluded |  |
| American (2) | 0 | NA |  | NA |  | 0 | NA |  | Excluded |  |
| **Educational level** | | | | | | | | | | |
| Elementary (3) | 3 (100) | 0.89E+10 | 0.005 | Excluded | NA | 3 (100) | 0.05E+10 | 0.009 | 0.01E+10 | 0.031 |
| Secondary (13) | 2 (15.4) | 1 |  | Excluded |  | 10 (76.9) | 1 |  | 1 |  |
| Higher (35) | 3 (8.6) | 0.516 (0.076-3.502) |  | Excluded |  | 12 (34.3) | 0.323 (0.072-1.440) |  | 0.071 (0.012-0.405) |  |
| Unknown (27) | 5 (18.5) | 1.250 (0.208-7.505) |  | Excluded |  | 14 (51.9) | 0.157 (0.036-0.679) |  | 0.158 (0.028-0.897) |  |
| **IDU** | | | | | | | | | | |
| No (76) | 12 (15.8) | 1 | 0.270 | NA | NA | 38 (50) | 1 | 1 | NA | NA |
| Yes (2) | 1 (50) | 5.333 (0.312-91.243) |  | NA |  | 1 (50) | 1 (0.060-16.577) |  | NA |  |
| **Recreational drug users** | | | | | | | | |  |  |
| No (18) | 1 (5.6) | 1 | 0.278 | NA | NA | 11 (61.1) | 1 | 0.438 | NA | NA |
| Yes (54) | 11 (20.4) | 4.349 (0.321-36.330) |  | NA |  | 26 (48.1) | 0.591 (0.199-1.753) |  | NA |  |
| Unknown (6) | 1 (16.7) | 3.4 (0.179-64.682) |  | NA |  | 2 (33.3) | 0.318 (0.046-2.223) |  | NA |  |
| **Sex partners in previous year** | | | | | | | | | | |
| <=5 (26) | 4 (15.4) | 1 | 0.710 | NA | NA | 16 (61.5) | 1 | 0.417 | NA | NA |
| 6-10 (12) | 2 (16.7) | 1.1 (0.172-7.029) |  | NA |  | 6 (50) | 0.625 (0.157-2.485) |  | NA |  |
| 11-20 (10) | 1 (10.0) | 0.611 (0.060-6.246) |  | NA |  | 3 (30) | 0.268 (0.056-1.283) |  | NA |  |
| 21-30 (2) | 0 | NA |  | NA |  | 0 | NA |  | NA |  |
| 31-40 (5) | 1 (20.0) | 1.375 (0.120-15.721) |  | NA |  | 2 (40) | 0.417 (0.059-2.946) |  | NA |  |
| 41-50 (5) | 1 (20.0) | 1.375 (0.120-15.721) |  | NA |  | 2 (40) | 0.417 (0.059-2.946) |  | NA |  |
| >50 (13) | 4 (30.8) | 2.444 (0.499-11.965) |  | NA |  | 8 (61.5) | 1 (0.254-3.929) |  | NA |  |
| Unknown (5) | 0 | NA |  | NA |  | 2 (40) | 0.417 (0.059-2.946) |  | NA |  |
| **Anatomical localization** | | | | | | | | | | |
| Urethra (30) | 4 (13.3) | 1 | 0.359 | NA | NA | 11 (36.7) | 1 | 0.111 | NA | NA |
| Rectum (35) | 8 (22.9) | 1.926 (0.517-7.177) |  | NA |  | 19 (54.3) | 2.051 (0.757-5.558) |  | NA |  |
| Pharynx (13) | 1 (7.7) | 0.542 (0.055-5.379) |  | NA |  | 9 (69.2) | 3.886 (0.966-15.640) |  | NA |  |
| **Symptomatology** | | | | | | | | | | |
| No (32) | 6 (18.8) | 1 | 0.131 | NA | NA | 20 (62.5) | 1 | 0.074 | Excluded | NA |
| Yes (45) | 6 (13.3) | 0.607 (0.194-2.294) |  | NA |  | 18 (40.0) | 0.400 (0.158-1.015) |  | Excluded |  |
| Unknown (1) | 1 (100) | 0.70E+10 |  | NA |  | 1 (100) | 0.10E+10 |  | Excluded |  |
| **HIV status** | | | | | | | | | | |
| - (40) | 4 (10.0) | 1 | 0.071 | Excluded | NA | 19 (47.5) | 1 | 0.791 | NA | NA |
| + (35) | 9 (25.7) | 3.115 (0.865-11.219) |  | Excluded |  | 18 (51.4) | 1.170 (0.472-2.902) |  | NA |  |
| Unknown (3) | 0 | NA |  | Excluded |  | 2 (66.7) | 2.211 (0.185-26.380) |  | NA |  |
| **Other STI different from Gonorrhoea** | | | | | | | | | | |
| No (54) | 7 (13.0) | 1 | 0.200 | NA | NA | 29 (53.7) | 1 | 0.326 | NA | NA |
| Yes (24) | 6 (25.0) | 2.238 (0.662-7.568) |  | NA |  | 10 (41.7) | 0.616 (0.233-1.627) |  | NA |  |
| Other STI in the previous year | | | | | | | | | | |
| No (46) | 4 (8.7) | 1 | 0.020 | 1 | 0.024 | 19 (41.3) | 1 | 0.045 | 1 | 0.004 |
| Yes (31) | 9 (29.0) | 4.295 (1.187-15.539) |  | 5.108 (1.242-21.005) |  | 20 (64.5) | 2.584 (1.008-6.622) |  | 6.243 (1.797-21.686) |  |
| Unknown (1) | 0 | NA |  | NA |  | 0 | NA |  | NA |  |
| **Number of anatomical sites with gonorrhoeae** | | | | | | | | | | |
| 1 (64) | 11 (17.2) | 1 | 0.003 | Excluded | NA | 26 (40.63) | 1 | 0.001 | 1 | 0.016 |
| 2 (12) | 0 | NA |  | Excluded |  | 11 (91.7) | 16.077 (1.955-132.211) |  | 27.589 (2.865-265.686) |  |
| 3 (2) | 2 (100) | 0.78E+10 |  | Excluded |  | 2 (100) | 0.24E+10 |  | 6.243 |  |

n: number of *N. gonorrhoeae* strains isolated from MSM.

MIC: minimum inhibitory concentration.

Tetracycline (I+R) - EUCAST: Tetracycline decreased susceptibility (MIC > 0.5mg/L) according to EUCAST breakpoints.

Tetracycline (I+R) - CLSI: Tetracycline decreased susceptibility (MIC > 0.25mg/L) according to CLSI breakpoints.

N: number of tetracycline decreased susceptible isolates.

OR: odds ratio.

CI: confident interval.

aOR: adjusted OR.

NA: not applicable.

Excluded: variable excluded by backward method in logistic regression analysis.
